# The differential effects of FTY720 on functional recovery and infarct size following myocardial ischaemia/ reperfusion

**DOI:** 10.5830/CVJA-2016-039

**Published:** 2016

**Authors:** Derick van Vuuren, Erna Marais, Sonia Genade, Amanda Lochner

**Affiliations:** Division of Medical Physiology, Department of Biomedical Sciences, Faculty of Medicine and Health Sciences, Stellenbosch University, Tygerberg, South Africa; Division of Medical Physiology, Department of Biomedical Sciences, Faculty of Medicine and Health Sciences, Stellenbosch University, Tygerberg, South Africa; Division of Medical Physiology, Department of Biomedical Sciences, Faculty of Medicine and Health Sciences, Stellenbosch University, Tygerberg, South Africa; Division of Medical Physiology, Department of Biomedical Sciences, Faculty of Medicine and Health Sciences, Stellenbosch University, Tygerberg, South Africa

**Keywords:** Functional recovery, FTY720, ischaemia/reperfusion injury, infarct size, myocardial, working heart perfusion

## Abstract

**Aim:**

The aim of this study was to evaluate the effects of the sphingosine analogue, FTY720 (Fingolimod), on the outcomes of myocardial ischaemia/reperfusion (I/R) injury.

**Methods:**

Two concentrations of FTY720 (1 or 2.5 μM) were administered either prior to (PreFTY), or following (PostFTY) 20 minutes’ global (GI) or 35 minutes’ regional ischaemia (RI) in the isolated, perfused, working rat heart. Functional recovery during reperfusion was assessed following both models of ischaemia, while infarct size (IFS) was determined following RI.

**Results:**

FTY720 at 1 μM exerted no effect on functional recovery, while 2.5 μM significantly impaired aortic output (AO) recovery when administered prior to GI (% recovery: control: 33.88 ± 6.12% vs PreFTY: 0%, n = 6–10; p < 0.001), as well as before and after RI (% recovery: control: 27.86 ± 13.22% vs PreFTY: 0.62%; p < 0.05; and PostFTY: 2.08%; p = 0.0585, n = 6). FTY720 at 1 μM administered during reperfusion reduced IFS [% of area at risk (AAR): control: 39.89 ± 3.93% vs PostFTY: 26.56 ± 4.32%, n = 6–8; p < 0.05), while 2.5 μM FTY720 reduced IFS irrespective of the time of administration (% of AAR: control: 39.89 ± 3.93% vs PreFTY: 29.97 ± 1.03%; and PostFTY: 30.45 ± 2.16%, n = 6; p < 0.05).

**Conclusion:**

FTY720 exerted divergent outcomes on function and tissue survival depending on the concentration administered, as well as the timing of administration.

## Aims

In 2008 the WHO reported that non-communicable diseases (NCDs), including cardiovascular disease, are the leading causes of death globally.^1^ South Africa, as well as the broader African region, is no exception, with recent research indicating the prevalence of NCDs and cardiovascular disease.^2^-^5^ Ischaemic heart disease has been identified as a major contributor to global morbidity and mortality rates^6^ in a trend set to continue, irrespective of affluence.^7^,^8^

The current clinical approach to myocardial ischaemia is to limit the duration of ischaemia by re-establishing perfusion of the affected tissue as fast as possible.^9^,^10^ The first description of ischaemic preconditioning^11^ however also exposed the innate ability of the heart to increase its resistance to ischaemia/reperfusion (I/R) injury. The fact that interventions can also be applied in conjunction with reperfusion to enhance the beneficial effects of reperfusion proves that (1) a degree of damage is imparted by reperfusion per se, a phenomenon that is known as reperfusion injury;^12^,^13^ and (2) the modulation of intracellular events inside the cardiomyocyte can protect the heart over and above the beneficial effect of rapid reperfusion.^13^ This is known as cardioprotection.

Research has revealed several well-defined intracellular signalling pathways associated with cardioprotection, including the reperfusion injury salvage kinase (RISK)^14^ and survivor activating factor enhancement (SAFE)^15^ pathways. These, and others, have been extensively reviewed elsewhere.^16^,^17^ Two molecules that have been implicated in the mediation of cardioprotection is the sphingolipid, sphingosine and its phosphorylated metabolite, sphingosine-1-phosphate (S1P).^18^,^19^ It has been shown that sphingosine and S1P can elicit cardioprotection through the activation of several of the known cardioprotective pathways.^20^-^24^

FTY720 (also known as Fingolimod), a derivative of a metabolite of a fungal species that has long been associated with medicinal effects in Chinese folk medicine,^25^ is a structural analogue of sphingosine. As such, it is metabolised in a similar fashion to sphingosine in that; it easily traverses the cell membrane to be phosphorylated intracellularly by sphingosine kinase 2 (SK2). The phosphorylated FTY720 (P-FTY720) then exits the cell to bind to a sphingosine-1-phosphate (S1P) receptor. Five receptors have been identified, of which four can interact with P-FTY720: S1P1, 3, 4 and 5.^26^,^27^

Since S1P and sphingosine have been associated with a reduction in the myocardial damage caused by I/R,^20^-^14^ several researchers have turned their attention to the potential benefits of FTY720 within this setting, with mixed success. Especially three endpoints have been addressed by the current body of research: rhythmicity, cell death/survival and functional ability following I/R. FTY720 appears to exert an effect on rhythmicity, however, the results obtained are controversial. Egom and co-workers^28^ reported that FTY270 reduced the occurrence of rhythmic disturbances post-I/R in an ex vivo rat heart, as well as in a sino-atrial node preparation. These beneficial effects were however absent in an in vivo rat model, where FTY720 administered during reperfusion proved detrimental due to an increased occurrence of tachycardia and ventricular fibrillation.^29^

Similarly, the effects of FTY720 on cell death are controversial; some researchers have reported that it decreases infarct size (IFS)^30^ and increases cell viability,^31^ while others have found no effect on IFS and cell survival.^29^,^32^ There seems to be more consensus regarding function following I/R. FTY720 given at the onset of reperfusion increases functional ability.^30^,^32^ The effect of acute pre-ischaemic administration of FTY720 to the heart has not yet been described.

Clinical interest in FTY720 has however not centred on its postulated cardioprotective effects, but rather on its immunosuppressant effects, which led to the approval of a commercial form of the drug for use as an oral treatment for multiple sclerosis (MS).^33^,^34^ One of the proposed explanations for the mechanism by which FTY720 suppresses the autoimmune response associated with MS is by binding to S1P1 in the lymphoid tissue. This initiates a paradoxical reduction in the effects linked to S1P1 activation, including the egress of lymphoid cells from the lymph nodes. This ‘functional antagonism’, as Brinkmann^26^ puts it, can be explained by the down-regulation of S1P1 receptors due to sustained activation by P-FTY720. The end-effect is the specific suppression of lymphocyte release from the lymph tissue.^25^-^27^

In addition to its immuno-modulatory effects, FTY720 has also received attention as a possible tumour suppressor.^35^ Studies have shown that FTY720 induces cell death in cancerous cells, while not eliciting any toxic effects in other tissues.^35^-^37^ One of the proposed mechanisms by which FTY720 induces cell death in these cells entails the FTY720-mediated activation of the serine/threonine protein phosphatase, protein phosphatase 2A (PP2A), which, when activated, favours the de-phosphorylation and inactivation of pro-survival proteins such as protein kinase B (PKB/Akt), extracellular signal-regulated kinase p42/p44 (ERK p42/p44), Bad and others.^36^-^39^

FTY720 is therefore a drug with both existing and potential clinical applications in divergent fields: MS, cancer and cardioprotection following I/R, although the precise nature of its influence in the latter context is still controversial. Consequently it is important to clarify its effects on the heart since, on one hand, FTY720 could present as a clinically feasible drug for the treatment of myocardial I/R injury, while on the other hand, it also has clinical potential in other scenarios, making it important to better illuminate the ‘off-target’ effects of this drug on organs such as the heart.

The controversies associated with FTY720 administration in the context of myocardial I/R injury highlight questions regarding the effects of different doses of the drug, administered at different time points relative to sustained ischaemia, on different endpoints associated with I/R injury. The aim of this study was therefore to investigate and describe the effects of FTY720 administration at two different concentrations (1 and 2.5 μM) prior to ischaemia or at the onset of reperfusion in two different models of ischaemia (20 minutes’ global ischaemia and 35 minutes’ regional ischaemia) on different endpoints (functional recovery and IFS) in the isolated, working rat heart model. In view of the immunomodulatory effects of FTY720, the isolated heart preparation allows for the study of direct cardiac effects independent of systemic, in this case specifically, immune interactions and effects.^40^

Regional ischaemia (RI) of the isolated heart is an accepted model of ischaemia of only a portion of the left ventricle, thereby simulating myocardial infarction. Global ischaemia (GI) on the other hand is of scientific interest, as it is well characterised and commonly used in basic research studies requiring relatively large amounts of homogenous tissue where biochemical analysis of tissue is required, or in instances where functional recovery is the primary endpoint of interest, for example in studies investigating stunning.^40^,^41^

## Methods

Male Wistar rats were allowed free access to food and water prior to experimentation. Rats weighing between 250 and 350 g were anaesthetised by intraperitoneal injection of 60 mg pentobarbital per rat. All experimental protocols were approved by the Animal Ethics committee of the University of Stellenbosch (Faculty of Medicine and Health Sciences) and were executed in accordance with the revised South African National Standard for the care and use of laboratory animals for scientific purposes (SABS, SANS 10386, 2008).

**Fig. 1.**
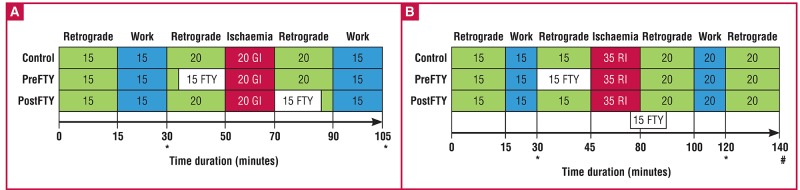
Isolated working rat heart perfusion protocols used to investigate the effects of different concentrations of FTY720, administered at different time points relative to sustained ischaemia, in different models of ischaemia and their respective endpoints. FTY720 (1 μM or 2.5 μM) was administered for a period of 15 minutes either immediately prior to sustained ischaemia (PreFTY), or directly at the onset of reperfusion (PostFTY). Two models of ischaemia were used: (A) 20 minutes’ global ischaemia (GI), followed by a total of 35 minutes’ reperfusion; and (B) 35 minutes’ regional ischaemia (RI), followed by 60 minutes’ reperfusion. In the RI model, the reperfusion administration of FTY720 already commenced during the final five minutes of RI. Functional recovery was measured in both groups at the end of the last episode of work during reperfusion. Infarct size (IFS) was the primary endpoint for this model of ischaemia. *Determination of functional ability both pre- and post ischaemia. #Determination of infact size.

For all experimentation, the isolated, working rat heart preparation was used as described previously.^42^ Following the establishment of sufficient sedation, the heart was rapidly removed and mounted by cannulation of the aorta, where after it was exposed to a perfusion protocol containing periods of both retrograde, as well as work-mode perfusion, as shown in Fig. 1.

Cardiac temperature was monitored throughout using a thermal probe inserted into the coronary sinus and maintained at a temperature of 36.5°C. Functional performance of the hearts was determined by the timed measurement of coronary flow (CF) during retrograde perfusion, as well as aortic output (AO) and CF during work mode. A pressure transducer (Viggo Spectromed) inserted into the aortic cannula was used to determine heart rate, as well as intra-aortic pressure. Aortic pressure and cardiac output (CO = AO + CF) were used to determine left ventricular work performance, as described by Kannengieser et al.^43^ Following a suitable period of stabilisation, the hearts were exposed to either 20 minutes of GI, or 35 minutes of RI as described in the next section.

FTY720 was obtained from Cayman Chemical (product number 10006292; Cayman Chemical, MI, USA). After dissolving it in dimethyl sulfoxide (DMSO), it was administered to the isolated hearts at a concentration of either 1 or 2.5 μM. The DMSO never exceeded a final concentration of 0.03% (vol/vol), i.e. 0.004 M. This concentration was lower than those reported to be associated with toxicity^44^ or changes in cardiac function.^45^

Both concentrations of FTY720 were administered to the isolated rat heart for a period of 15 minutes directly prior to (PreFTY) sustained ischaemia or at the onset of reperfusion (PostFTY) (Fig. 1). Global ischaemia (GI) entailed the complete cessation of perfusion of the heart for a period of 20 minutes, at a temperature of 36.5°C. Function during reperfusion was expressed relative to pre-ischaemic values and served as an endpoint for the damage caused by ischaemia. RI was initiated by occluding the left anterior descending coronary artery using a silk suture to ensnare the proximal section of the artery and closing it with two interlocking pieces of plastic tubing, thereby rendering the tissue distal to the occlusion ischaemic [the area at risk (AAR)], while the remainder of the heart still received adequate perfusion [the viable area (VA)].

Following 35 minutes of RI, at a maintained temperature of 36.5°C, the AAR was reperfused by opening the suture. Following RI, FTY720 administration was initiated five minutes before the end of ischaemia and progressed for the first 10 minutes of reperfusion. Both the extent of infarct development as well as functional recovery were used to assess the effects of this ischaemic stress.

Hearts were reperfused for a period of 35 minutes following 20 minutes’ GI, and for 60 minutes following 35 minutes’ RI. Although many researchers in the field prefer longer periods of reperfusion following RI, previous work in our laboratory has shown that shorter periods of reperfusion did not influence the relative degree of IFS development between groups.^46^,^47^ Post-ischaemic function was assessed at 35 minutes’ reperfusion following 20 minutes’ GI, and 40 minutes’ reperfusion following RI.

Infarct size was determined as previously described.^48^,^49^ Briefly, following the application of RI and a suitable period of reperfusion, the suture surrounding the left coronary artery was re-occluded and the heart was infused with 0.5% Evans blue dye, administered through the aortic cannula. This then delineated the VA (which received adequate perfusion throughout the protocol) from the AAR (the portion of the heart that was exposed to ischaemia, including infarcted tissue).^50^,^51^

**Fig. 2.**
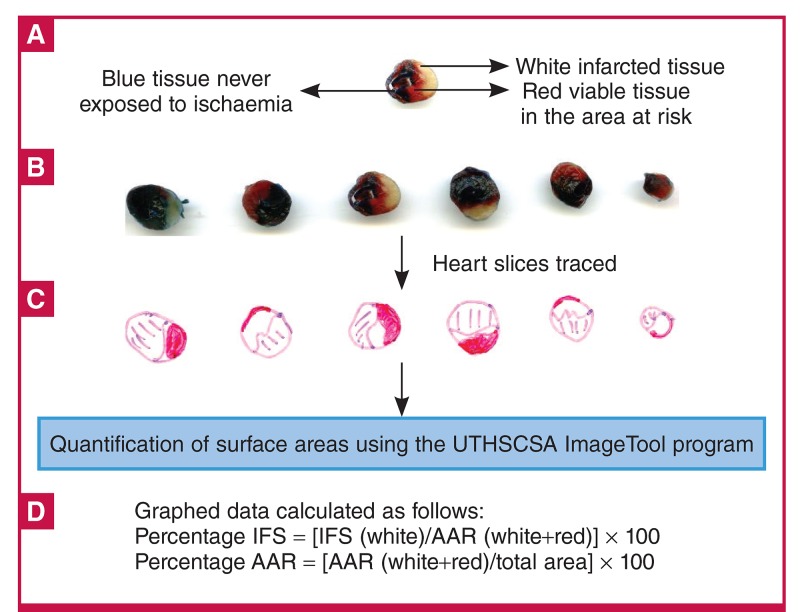
Graphic scheme illustrating the determination of infarct size (IFS) using Evans blue dye and triphenyltetrazolium chloride (TTC) staining. A, B. Evans dye is retrogradely injected through the aorta of the heart to distinguish between tissue that has received adequate perfusion (perfusing blue) and tissue exposed to ischaemia. This ischaemic area is also called the area at risk (AAR). It contains both infarcted tissue (white) and viable tissue, which stains red in a reaction with TTC. These three zones are then traced on an overlaying transparency (C), which is then scanned and the different areas determined using planimetry software. D. The data that is finally used for statistical comparison is the infarct size expressed as a percentage of the AAR, as well as the AAR expressed relative to the total surface area. This latter parameter is an indication of the degree of coronary occlusion.

The heart was then promptly removed from the perfusion apparatus and frozen at –20°C for later analysis. After no more than five days, the frozen ventricles were cut into slices of approximately 2 mm in thickness and stained with 1% w/v triphenyltetrazolium chloride (TTC) in a phosphate buffer (pH 7.4) at room temperature. Triphenyltetrazolium chloride (TTC) stains viable tissue a brick-red colour through its reaction with active dehydrogenases.^51^,^52^ After 15 minutes, the heart slices were fixed in a 10% v/v formaldehyde solution. The final result was slices of heart tissue stained blue (VA), red (viable tissue in the AAR) and white (infarcted tissue in the AAR) (Fig. 2).

The surface areas of these zones were quantified using the UTHSCSA ImageTool program (developed at the University of Texas Health Science Center at San Antonio, Texas, which is available from the internet at http://ddsdx.uthscsa.edu/dig/itdesc.html) and the data for all the individual slices were added together for each heart. For comparison of the data, IFS is expressed relative to the AAR, while the degree of occlusion is indicated by the AAR relative to the total area (AAR + VA). For an overview of the determination of IFS in pre-clinical experimental models see Csonka et al.^51^

## Statistical analysis

Animals were randomly assigned to different experimental groups on the day of experimentation. Where functional recovery was assessed as the primary endpoint (i.e. after 20 minutes’ GI) data were collected from a minimum of four to a maximum of 10 rats per group. Infarct size was compared between groups containing a minimum of six rats per group. All statistical analyses were done using Graphpad Prism (version 6.01). For all comparisons (unless otherwise specified) a one-way analysis of variance (ANOVA) was performed, followed by Dunnett’s test to compare all relevant experimental groups to control alone. A p-value of less than 0.05 was considered statistically significant.

## Results

## The effect of FTY720 in a model of 20 minutes’ GI

Isolated, working rat hearts exposed to 20 minutes’ GI were treated with either 1 or 2.5 μM FTY720, either prior to sustained ischaemia or immediately at the onset of reperfusion (Fig. 1). All functional data recorded in this study are shown in Table 1. Baseline functional measurements of the hearts, prior to both FTY720 administration and ischaemia, are shown in Table 2. There were no significant differences in the basal capacity of any of the groups compared to the control.

**Table 1:** Functional parameters of isolated, perfused rat hearts recorded at baseline (i.e. prior to both the administration of FTY720, as well as exposure to sustained ischaemia) and at the end of work-mode reperfusion (following sustained ischaemia). Reperfusion function was compared to baseline function using a paired t-test

**	*Coronary flow† (ml/min)*		*Aortic output (ml/min)*		*Cardiac output (ml/min)*		*Total work (mW)*		*Heart rate (beats/min)*		*Systolic pressure (mmHg)*		**
*Group*	*Baseline*	*Post-ischaemic*	*Baseline*	*Post-ischaemic*	*Baseline*	*Post-ischaemic*	*Baseline*	*Post-ischaemic*	*Baseline*	*Post-ischaemic*	*Baseline*	*Post-ischaemic*	*n*
20 minutes’ global ischaemia													
Control	8.45 ± 0.75	7.50 ± 0.80	44.20 ± 3.41	16.00 ±3.75*	60.55 ± 4.10	28.35 ± 4.29*	13.17 ± 1.22	5.92 ± 1.22#	317.4 ± 19.8	263.4 ± 13.5	90.25 ± 3.75	78.88 ± 3.84^@^	5–10
1 μM FTY720													
PreFTY	9.13 ± 1.13	6.31 ± 0.43@	47.13 ± 4.11	13.50 ± 3.62*	63.81 ± 4.78	24.25 ± 4.83*	14.41 ± 0.56	5.94 ± 1.09$	278.6 ± 18.4	255.0 ± 15.9	96.25 ± 4.05	82.25 ± 3.39	5–8
PostFTY	6.70 ± 0.47	12.20 ± 0.93*	51.50 ± 1.78	14.00 ± 3.66*	66.39 ± 2.45	23.72 ± 5.45*	14.02 ± 0.74	4.96 ± 1.24*	267.0 ± 10.2	189.0 ± 37.1	94.89 ± 3.01	72.11 ± 14.01	9–10
2.5 μM FTY720													
PostFTY	6.50 ± 0.52	6.58 ± 0.37	41.00 ± 5.21	0.0 ± 0.0^$^	55.25 ± 6.48	5.92 ± 0.45^$^	13.98 ± 0.41	0.40 ± 0.40*	266.8 ± 23.5	24.3 ± 24.3^#^	101.7 ± 4.78	16.00 ± 16.00^#^	4–6
PostFTY	5.50 ± 0.49	9.57 ± 0.98#	44.29 ± 2.88	8.21 ± 1.83^$^	58.93 ± 4.81	24.00 ± 4.73*	11.54 ± 1.09	4.60 ± 0.92*	282.1 ± 15.2	259.4 ± 9.6	88.71 ± 5.47	77.14 ± 3.23^@^	6–7
35 minutes’ regional ischaemia													
Control	9.00 ± 0.52	9.27 ± 0.46	48.33 ± 3.56	14.33 ± 6.82^#^	62.83 ± 4.34	29.83 ± 7.09^#^	13.46 ± 1.13	5.60 ± 1.49^#^	292.7 ± 10.3	277.5 ± 8.0^@^	96.00 ± 1.88	81.33 ± 2.72^#^	6
1 μM FTY720													
PreFTY	8.92 ± 0.45	9.50 ± 1.71	55.50 ± 1.63	9.50 ± 3.38*	69.08 ± 3.98	19.75 ± 4.52^$^	15.46 ± 0.63	3.98 ± 0.55*	274.7 ± 9.9	216.5 ± 38.8	96.50 ± 1.50	76.33 ± 5.40^#^	6
PostFTY	10.00 ± 0.82	12.07 ± 1.14	44.00 ± 4.30	2.57 ± 1.49^$^	58.14 ± 5.49	14.16 ± 3.59^#^	11.67 ± 1.13	2.31 ± 0.74^$^	285.6 ± 15.5	205.1 ± 40.8^@^	90.29 ± 1.11	59.29 ± 12.03^@^	7
2.5 μM FTY720													
PreFTY	10.25 ± 0.54	9.42 ± 0.27	47.00 ± 3.53	0.33 ± 0.33*	63.17 ± 4.39	12.08 ± 4.09^$^	13.02 ± 0.98	1.82 ± 0.67^$^	279.7 ± 17.4	146.3 ± 47.3	92.67 ± 1.20	45.50 ± 15.74^@^	6
PreFTY	9.67 ± 0.76	10.67 ± 0.76	52.33 ± 3.20	1.00 ± 1.00*	69.42 ± 3.94	9.42 ± 3.25^$^	14.62 ± 0.87	1.55 ± 0.58^$^	269.8 ± 13.2	156.0 ± 50.2	94.83 ± 1.08	48.33 ± 15.66^@^	6

^†^As measured during retrograde perfusion; *p < 0.0001 versus baseline; $p < 0.001; #p < 0.01 versus baseline; @p < 0.05 versus baseline

**Table 2:** Baseline functional ability of isolated rat hearts prior to exposure to both 20 minutes’ global ischaemia (GI), as well as treatment with FTY720 (1 or 2.5 μM) either before sustained ischaemia or during initial reperfusion

**	*Aortic output*	*Cardiac output*	*Total work*	**
*Group*	*(ml/min)*	*(ml/min)*	*(mW)*	*Number*
Control	44.20 ± 3.41	60.55 ± 4.10	13.17 ± 1.22	5–10
1 μM FTY720				
PreFTY	47.13 ± 4.11	63.81 ± 4.78	14.41 ± 0.56	5–8
PostFTY	51.50 ± 1.78	66.39 ± 2.45	14.02 ± 0.74	9–10
2.5 μM FTY720				
PreFTY	41.00 ± 5.21	55.25 ± 6.48	13.98 ± 0.41	4–6
PostFTY	44.29 ± 2.88	58.93 ± 4.81	11.54 ± 1.09	6–7

**Fig. 3.**
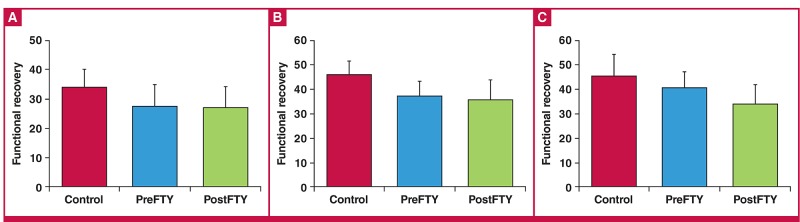
The effect of FTY720 (1 μM) administered either immediately before sustained ischaemia (PreFTY), or for the first 15 minutes of reperfusion (PostFTY), on functional recovery in isolated hearts exposed to 20 minutes’ GI, followed by 35 minutes’ reperfusion. Functional recovery was assessed as post-ischaemic (A) aortic output (AO); (B) cardiac output (CO); and (C) total work expressed as a percentage of pre-ischaemic values, n = 5–10.

**Fig. 4.**
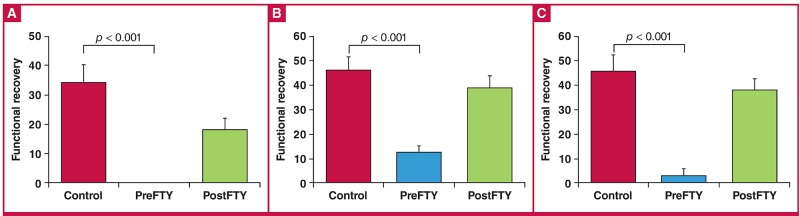
The effect of FTY720 (2.5 μM) administered as either pre-treatment (PreFTY) or immediately following ischaemia (PostFTY) on functional recovery. Pre-ischaemic administration of FTY720 was associated with a profound suppression of functional recovery in terms of (A) aortic output, (B) cardiac output, and (C) total work, n = 4–10.

Administration of FTY720 at a dose of 1 μM had no effect on functional recovery (Fig. 3), irrespective of whether it was administered before ischaemia or during early reperfusion. Similar to the effects associated with 1 μM, reperfusion administration of 2.5 μM FTY720 did not exert any significant effects on post-ischaemic function (Fig. 4).

Surprisingly, at a dose of 2.5 μM, FTY720 administered as a pre-treatment potently suppressed all functional recovery (Fig. 4), as evident by the fact that of the six hearts pre-treated with FTY720, only one recovered sufficiently to generate recordable total work data (aortic output: control: 33.88 ± 6.12% vs PreFTY: 0%, n = 6–10; p < 0.001; cardiac output: control: 45.94 ± 5.57% vs PreFTY: 12.20 ± 2.68%, n = 6–10; p < 0.001; and total work: control: 45.67 ± 8.98% vs PreFTY: 2.79%, n = 4–5; p 7lt; 0.01).

In view of this severe suppression of post-ischaemic function, we also analysed heart rate (percentage recovery: control: 85.00 ± 6.11% vs PreFTY: 10.53%, n = 6–8; p < 0.001) as well as systolic pressure (percentage recovery: control: 88.13 ± 4.45% vs PreFTY: 15.10%, n = 6–8; p < 0.001). We found both to be significantly reduced in the FTY720 pre-treatment group.

**Fig. 5.**
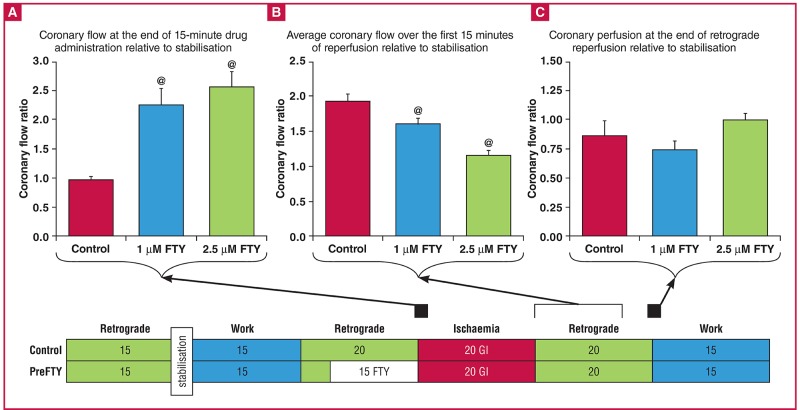
The effect of pre-treatment with FTY720 on coronary flow (CF) in a model of global ischaemia. Coronary flow was collected during retrograde perfusion at the end of stabilisation, the end of drug administration and every two minutes for the first 15 minutes of reperfusion, as well as at the end of retrograde reperfusion. For comparison purposes, the CF of each time point was expressed relative to the stabilisation values of that group. FTY720 increased CF during administration of the drug, but reduced CF during initial reperfusion.@p < 0.05 vs control, n = 3–11.

**Fig. 6.**
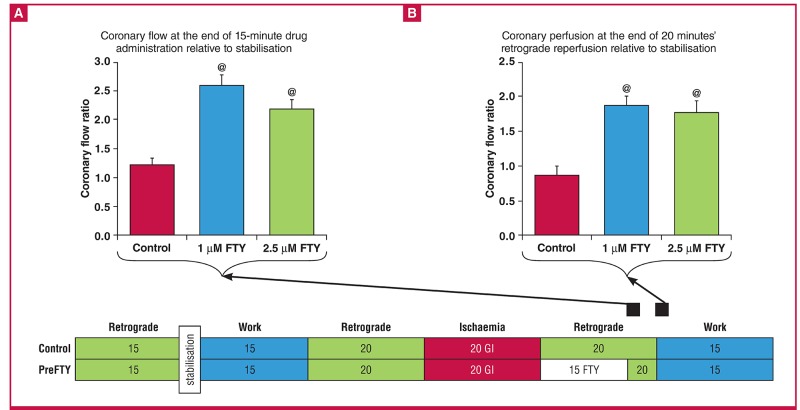
The effect of the reperfusion administration of FTY720 on coronary flow (CF). FTY720 was associated with an increase in CF, which was still evident five minutes after the cessation of drug administration.@p < 0.01 vs control, n = 4–11.

During the perfusion experiments we noticed that FTY720 elicited a profound increase in coronary flow (CF) (Figs 5, 6). Expression of CF at the end of pre-ischaemic drug administration relative to retrograde perfusion stabilisation values (Fig. 5) shows that both doses increased CF when administered prior to ischaemia [control: 0.95 ± 0.05 arbitrary units (AU) vs PreFTY (1 μM): 2.25 ± 0.27 AU and PreFTY (2.5 μM): 2.56 ± 0.27 AU, n = 5–8; p < 0.01]. Expression of reperfusion CF relative to pre-ischaemic stabilisation CF reveals a similar trend when FTY720 was administered at the onset of reperfusion [Fig. 6; control: 1.21 ± 0.11 AU vs PostFTY (1 μM): 2.59 ± 0.18 AU and PostFTY (2.5 μM): 2.19 ± 0.15 AU, n = 4–9; p < 0.01].

Surprisingly, the vasodilatory effect of FTY720 pre-treatment (Fig. 5) was replaced by a reduction in CF during the first 15 minutes of reperfusion relative to the control [CF expressed relative to retrograde stabilisation: control: 1.94 ± 0.17 AU vs PreFTY (1 μM): 1.60 ± 0.08 AU and PreFTY (2.5 μM): 1.14 ± 0.07 AU, n = 3–5; p < 0.05]. This effect was however transient, with CF in the drug-treated groups returning to control levels after 20 minutes of retrograde perfusion (Fig. 5C).

## The effect of FTY720 in a model of 35 minutes’ RI

Although functional recovery is a useful endpoint, IFS is considered by many workers to be the gold standard for evaluating the effects of an intervention on I/R injury. We therefore also investigated the effects of FTY720 administration on IFS following 35 minutes of RI (Fig. 1). Although IFS was the primary endpoint for these experiments, functional recovery was also recorded.

Pre-ischaemic functional patency of the hearts used for these experiments is shown in Table 3. None of the experimental groups differed from the control group prior to the administration of FTY720 and the onset of regional ischaemia.

**Table 3:** Baseline functional ability of isolated rat hearts prior to exposure to both 35 minutes’ regional ischaemia (RI), as well as treatment with FTY720 (1 or 2.5 μM) either before sustained ischaemia or during initial reperfusion

**	*Aortic output*	*Cardiac*	*Total work*	**
*Group*	*(ml/min)*	*(ml/min)*	*(mW)*	*Number*
Control	48.33 ± 3.56	62.83 ± 4.34	13.46 ± 1.13	6
1 μM FTY720				
PreFTY	55.50 ± 1.63	69.08 ± 3.98	15.46 ± 0.63	6
PostFTY	44.00 ± 4.30	58.14 ± 5.49	11.67 ± 1.13	7
2.5 μM FTY720				
PreFTY	47.00 ± 3.53	63.17 ± 4.39	13.02 ± 0.98	6
PostFTY	52.33 ± 3.20	69.42 ± 3.94	14.62 ± 0.87	6

**Fig. 7.**
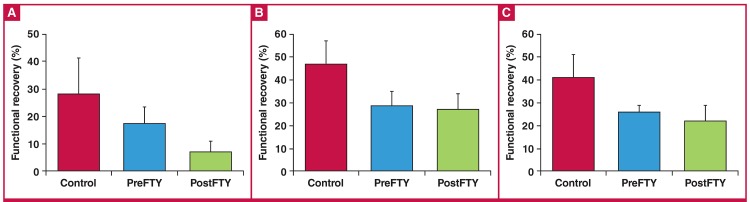
The effects of 1 μM FTY720, administered either before (PreFTY) ischaemia or at the onset of reperfusion (PostFTY), on functional recovery following 35 minutes of regional ischaemia (RI) in terms of (A) aortic output, (B) cardiac output, and (C) total work, n = 6–8.

One of the advantages of a model of regional ischaemia is that the functional ability of the hearts after the index ischaemia can also be measured alongside the primary endpoint of IFS. Table 1 contains the functional values recorded at reperfusion. Similar to our GI experiments, 1 μM of FTY720 failed to elicit any effect on functional recovery following 35 minutes of RI (Fig. 7).

**Fig. 8.**
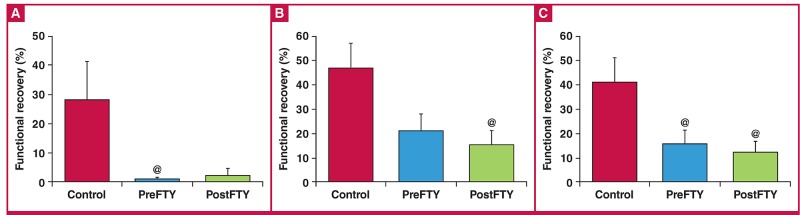
The effect of 2.5 μM FTY720 on functional recovery following 35 minutes’ regional ischaemia (RI) in terms of (A) aortic output, (B) cardiac output, and (C) total work. @p < 0.05 vs control, n = 6.

As was the case in the 20-minute GI model, FTY720 elicited a much more evident effect when administered at 2.5 μM (Fig. 8), showing a profound effect on functional recovery during reperfusion. Pre-treatment was associated with a reduction in aortic output (only one heart recovered sufficiently to generate AO: control: 27.86 ± 13.22% vs PreFTY: 0.62%, n = 6; p < 0.05) and work recovery (control: 40.74 ± 9.98% vs PreFTY: 15.07 ± 5.69%, n = 6; p < 0.05), while reperfusion administration significantly reduced cardiac output (control: 46.56 ± 10.25% vs PostFTY: 15.02 ± 5.38%, n = 6; p < 0.05) and work recovery (control: 40.74 ± 9.98% vs PostFTY: 11.84 ± 4.59%, n = 6; p < 0.05).

Although reperfusion treatment did not statistically reduce aortic output recovery, it is noteworthy that of the six hearts included in the group, only one recovered sufficiently to actually generate an aortic output. These profound inhibitory effects of reperfusion-administered FTY720 on post-ischaemic CO and work are especially intriguing in the light of the increase in CF shown to be associated with FTY720 administration (Fig. 6). These combined observations suggest a direct effect of FTY720 on contractility per se.

Since 2.5 μM FTY720 elicited such a strong detrimental effect on functional recovery, we also analysed two additional functional parameters: heart rate and systolic pressure. Unexpectedly, FTY720 treatment was not associated with a significant reduction in heart rate recovery (control: 95.00 ± 1.85% vs PreFTY: 56.92 ± 18.69% and PostFTY: 60.73 ± 19.46%, n = 6; p = NS) or systolic pressure recovery (control: 84.79 ± 2.69% vs PreFTY: 48.89 ± 17.03% and PostFTY: 50.96 ± 16.58%, n = 6; p = NS).

**Fig. 9.**
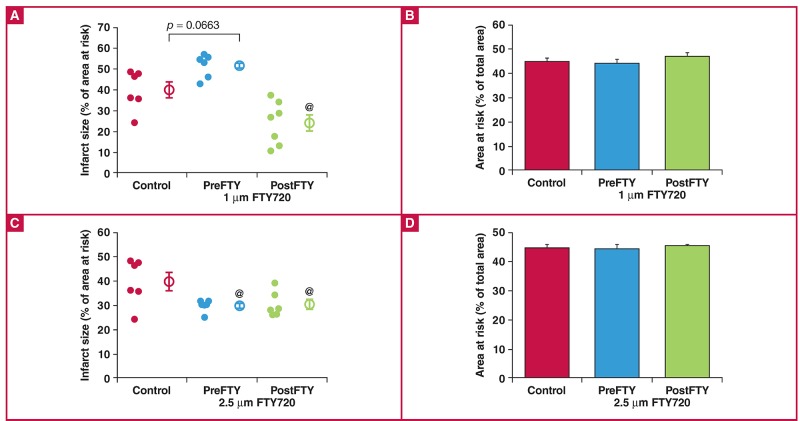
Effect of FTY720 on infarct size in hearts exposed to 35 minutes of regional ischaemia. At a dose of 1 μM (A), FTY720 reduced infarct size when administered at the onset of reperfusion, while pre-ischaemic treatment seemed to aggravate the ischaemic injury. At a higher dose of 2.5 μM, FTY720 however reduced infarct size (C) irrespective whether it was administered prior to ischaemia or during early reperfusion. Area at risk, an indication of the ischaemic intervention experimentally induced did not differ between any of the groups ((B) and (D)). @p < 0.05 vs control, n = 6–8.

Surprisingly, FTY720 exerted a considerably different effect on infarct size (Fig. 9), in contrast to the effects seen on functional recovery after regional ischaemia. Administration of 1 μM FTY720 as a pre-treatment showed a very strong tendency to increase IFS (control: 39.89 ± 3.93% vs PreFTY: 51.73 ± 2.36%, n = 6; p = 0.066), while administration during reperfusion limited the development of infarction (control: 39.89 ± 3.93% vs PostFTY: 23.96 ± 3.99%, n = 6–7; p < 0.05).

In contrast to this dependence on the time point of administration relative to sustained ischaemia, 2.5 μM FTY720 consistently reduced IFS (control: 39.89 ± 3.93% vs PreFTY: 29.97 ± 1.03% and PostFTY720: 30.45 ± 2.16%, n = 6; p < 0.05). The area at risk, relative to the total area, did not differ among any of the groups.

We are therefore left with these two disparate observations: on one hand, 1 μM FTY720 had no effect on post-ischaemic functional recovery and 2.5 μM reduced functional recovery, while on the other hand FTY720 caused a significant reduction in IFS, with the exception of the increase observed when 1 μM FTY720 was administered prior to ischaemia.

## Discussion

Recently there has been great interest in the sphingosine analogue, FTY720, mostly because of its immunomodulatory functions, but also because of its potential to stimulate similar pathways to sphingosine-1-phosphate. In this context, several researchers have investigated its potential to confer protection against myocardial ischaemia/reperfusion injury, however, with divergent results.

In this study we investigated the effects of two different concentrations of FTY720, administered prior to ischaemia or during initial reperfusion in two models of ischaemia with function and infarct size as endpoints. We found that 1 μM FTY720 exerted no effect on functional recovery regardless of the time of administration, although pre-treatment augmented infarction, while reperfusion treatment reduced IFS. Increasing the dose to 2.5 μM proved severely detrimental to functional recovery, although it was associated with an unexpected reduction in IFS.

FTY720 is a sphingosine analogue, therefore it can elicit similar effects to sphingosine and it can also be phosphorylated by intracellular SK2 to generate a phosphorylated form (P-FTY720) similar to S1P, which can exit the cell of origin and then bind on any one of the S1P receptors (except receptor 2).^53^ This leads to a conundrum when administering and experimenting with FTY720, which has also been reported by others,30,37 namely, are the effects observed due to FTY720 simulating sphingosine or P-FTY720 mimicking S1P?

Although we were unable to distinguish between these two forms of the drug in our experimental setup, there is an observation and an argument, which both point to a possible significant involvement of P-FTY720. First, a significant increase in coronary flow was observed during the administration of FTY720 at both dosages tested. This vasodilatory effect has been linked to the activation of S1P receptor 1 and/or 3 in the endothelium, which then recruits a PI3-kinase pathway, as well as a Ca2+-mediated mechanism to activate endothelial nitric oxide synthase (eNOS) to produce nitric oxide (NO), which ultimately facilitates vascular relaxation.^54^-^56^ Although the IC50 values for P-FTY720 binding to both S1P1 and S1P3 are extremely low (0.21 ± 0.17 nM for S1P1 and 5.0 ± 2.7 nM for S1P353), the observed vasodilation in our experimental hearts nonetheless implies the presence of P-FTY720 in the system. This possibility is further confirmed by the fact that the enzyme responsible for phosphorylating FTY270, SK2, is the predominant sphingosine kinase in the heart^57^,^58^ and is also activated by FTY720.^57^ It is therefore probable that the relatively high dose of FTY720 employed in this study would further stimulate FTY720 phosphorylation.

In this regard, Tölle and colleagues55 found that SK2 in human umbilical vein endothelial cells (HUVECs) and whole aortae converted FTY720 to P-FTY720 at a notable rate: 70% of 1 μM FTY720 was phosphorylated after two minutes, and 90% after 10 minutes. Taken together, it is highly likely that a large portion of the FTY720 administered in our study had been phosphorylated.

The S1P receptors are all G-coupled protein receptors, which, when activated, can induce the activation of several pathways associated with cardioprotection, such as protein kinase C (PKC), phosphatidylinositol-3-kinase (PI3-kinase) and protein kinase B (PKB/Akt).^20^-^24^,^59^ It is therefore no surprise that several researchers have investigated the ability of S1P to limit infarct size.^21^-^24^ Even unphosphorylated sphingosine has been associated with cardioprotection if administered concentrations are low enough (~0.4 μM22).

These observations have led to the question whether FTY720 can also be used to confer protection. In 2009, Hofmann and colleagues addressed this in an ex vivo rat heart preparation and found that although FTY720 increased post-ischaemic function, it failed to limit IFS,^32^ a result repeated in an in vivo model of ischaemia.^29^ In 2011, however, Egom et al.^31^ illustrated the ability of FTY720 to increase cell viability in isolated neonatal rat cardiomyocytes exposed to either simulated ischaemia or hypoxia. These observations were subsequently confirmed by Vessey and co-workers,^30^ who reported that 600 nM FTY720, administered as a post-conditioning intervention, exerted an infarct-sparing effect in an isolated mouse heart model exposed to global ischaemia.

Wang and colleagues^60^ administered FTY720 for a period of one or three weeks in a mouse model and found that this relatively chronic treatment regime also reduced IFS. Therefore our results agree with the latter set of findings that FTY720 is able to reduce infarct size. These results can probably be explained by the expected activation of S1P receptor-mediated pro-survival pathways and/or suppressed inflammation in the heart, as has been shown by others (although we did not assess the mechanism of protection).

The surprising exception was pre-treatment with 1 μM, which, at best, did not influence IFS, while not having an effect on postischaemic functional recovery. To our knowledge, this is the first study to have experimented with the acute administration of FTY720 prior to ischaemia, although both Hofmann et al.^29^ and Wang et al.^60^ administered the drug to their respective animal models for a substantial period of time (from one day once off to three weeks’ chronic treatment) prior to ischaemia. We did not investigate the mechanism by which FTY720 pre-treatment influences injury. We however propose that it can be explained, at least in part, by the concurrent activation of protein phosphatase 2A (PP2A). The concentration of FTY720 used in the present study was notably higher than those used by others. Egom et al.^28^ used as little as 25 nM, Hofmann et al.^32^ used 500 nM, while Vessey et al.^30^ used 600 nM. Our dose was based on work done on FTY720 as an activator of PP2A in the setting of cancer and cancerous cell lines.

In 2003, Matsuoka and colleagues^38^ reported that 8 μM FTY720 could suppress the phosphorylation of PKB/Akt, Bad and p70S6 kinase in T-cell leukaemia cells through the activation of PP2A. Others have also reported on the ability of FTY720 to activate PP2A in the range of 2.5 to 10 μM.^36^,^37^,^39^ Recently it was reported that FTY720 established this activation by binding to SET (Suvar3-9, enhancer of zeste, trithorax), an endogenous inhibitor of PP2A, thereby mediating the dissociation of SET from PP2A.^37^,^39^ This mechanism is however dependent on FTY720 not being phosphorylated, since P-FTY720 has the potential to phosphorylate SET through a Jak2-mediated pathway, thereby enhancing its PP2A inhibitory function.^37^ Others have however also shown that FTY720 can activate PP2A through the activation of a Pak1-mediated signalling pathway.^61^-^63^ It is therefore possible that the increase in infarct size that we observed in the 1-μM pre-treatment group was due to the activation of PP2A, which theoretically could suppress the phosphorylation-mediated pro-survival pathways, also co-activated by FTY720, which we have observed (unpublished data) and others have reported.^21^,^22^ ^30^-^32^,^63^

In contrast to this, in the 2.5-μM pre-treatment group, pro-survival activation probably dominated over PP2A activation, thereby inducing protection. As will be discussed in the following paragraphs, 2.5 μM FTY720 also induced a profound reduction in post-ischaemic functional ability, thereby reducing energy demand and also potentially contributing to a reduction in IFS.

Contrary to the infarct-sparing effects associated with FTY720 treatment in our study, we found that 1 μM exerted no effect on functional recovery, while 2.5 μM significantly suppressed post-ischaemic function in both the GI, as well as RI models. This is in contrast to Hofmann et al.^32^ and Vessey et al.,^30^ who both showed that FTY720 maintained functional ability after ischaemia at doses of 500 nM, 600 nM and 1 μM. It seems as if FTY720 has a dose-dependent effect, with an increase in FTY720 concentration being detrimental to postischaemic function. This was a surprising finding since, in the context of cancer research, it has been reported that relatively high doses of FTY720, administered chronically to mouse models, did not exert any toxic effects.^36^,^37^ Neither of these studies however investigated isolated heart performance or resistance to ischaemic stress.

The concentrations of FTY720 used in our study were also not as high as those of sphingosine, which have been shown by others to be detrimental: Suzuki and colleagues^64^ reported that 10 or 20 μM sphingosine induced apoptosis in several cell lines, while Karliner^18^ specified 5 μM of sphingosine as cardiotoxic. With regard to the phosphorylated form, Theilmeier and co-workers^59^ reported that a dose as high as 10 μM of S1P protected neonatal rat cardiomyocytes from apoptosis in a model of glucose and growth factor withdrawal. It is therefore unlikely that the FTY720 concentrations that we used were toxic. A possible explanation for our seemingly controversial results may be found in the perfusion model used, as well as the effects of S1P receptor stimulation on heart function per se.

Our experimental model differed from those used by others. Hofmann^32^ showed cardioprotection in retrogradely perfused rat hearts and human myocardial muscle strip preparations, while Vessey et al.^30^ used an isolated retrogradely perfused mouse heart model, with FTY720 administered as a postconditioning intervention (four cycles of five seconds’ ischaemia and reperfusion at the onset of reperfusion). We however utilised an isolated working rat heart preparation, which has an additional energy demand^65^ and free radical exposure^66^ associated with it. This more challenging setting than normal retrograde perfusion might explain the inability of FTY720 to protect functional capacity in our model, and even contribute to its loss.

Although not investigated by us, it has been reported by others that S1P receptor activation has the potential to suppress both heart rate, as well as contractility, largely through effects on intracellular calcium ion (Ca^2+^) dynamics. We propose that these mechanisms, at least in part, explain the observed detrimental effects of FTY720 on post-ischaemic function as follows. Heart tissue expresses S1P receptors 1, 2 and 3.^67^ Of these, it is only 1 and 3 that can be activated by P-FTY720.^53^ It has been reported that activation of these receptors in the heart, especially receptor 3, induces a reduction in heart rate^68^-^70^ through the activation of he inwardly rectifying atrial potassium ion channel (IKACh), thereby allowing an increased inward flux of potassium ions into the cell and hyperpolarising the sarcolemma.^70^,^71^

Activation of the S1P receptor 1 also exerts a negative inotropic effect, through a reduction in the availability of intracellular Ca^2+^ from the sarcoplasmic reticulum (SR) for the initiation of contraction. Two mechanisms have been shown to be involved in this inotropic effect: (1) similarly to the reduction in heart rate, activation of IKACh leads to the hyperpolarisation of the sarcolemma, leading to a subsequent reduction in action potential duration, which in turn implies a reduced influx of Ca^2+^ into the cardiomyocytes, thereby reducing the stimulus for the Ca^2+^-induced release of Ca^2+^ from the SR; (2) linking with the previous mechanism, S1P has been shown to reduce Ca2+ flux through the L-type Ca^2+^ channel, thereby also diminishing the potency of Ca^2+^-induced Ca^2+^ release. This reduction in flow through the L-type Ca^2+^ channel could be due to a Gi-mediated reduction in cyclic AMP levels, associated with the stimulation of the S1P receptors.^67^,^72^,^73^ Ironically, these same mechanisms that reduce intracellular Ca^2+^ levels might also limit Ca^2+^ overload during reperfusion, thereby contributing to the infarct-limiting effects of FTY720.

In view of the effect of S1P activation on heart rate (as discussed above), the divergent results that have been generated regarding the effect of FTY720 on rhythmicity,^28^,^29^ and the relevance of arrhythmia in the pathology of myocardial I/R injury, it is a limitation of this study that we did not include the incidence of arrhythmia in early reperfusion as an additional endpoint.

We speculate that the FTY720-mediated reduction in intracellular Ca^2+^ levels associated with a general reduction in functional ability, in combination with the dual stressor of I/R and work-mode perfusion could explain the severely detrimental effects of FTY720 on post-ischaemic functional recovery. It would therefore be interesting to assess the effects of acute FTY720 administration at these doses in hearts either not exposed to ischaemia and reperfusion, and/or not exposed to work-mode perfusion.

We are therefore confronted with two sets of disparate observations: FTY720 administration limited IFS, yet simultaneously suppressed functional recovery. Such a dissociation between changes in IFS and changes in functional recovery, as observed here, have been reported by us 44,74,75 and others.^76^-^78^ Although it is unexpected, it is therefore not without precedent or possible explanation through combination of the diverse effects of FTY720 on the heart.

FTY720, both unphosphorylated and phosphorylated, activates several cardioprotective pathways, possibly including components such as protein kinase A (PKA), protein kinase G (PKG), protein kinase C (PKC), PKB/Akt, ERK p42/p44 and Pak1.^21^,^22^,^30^-^32^,^63^ The robust activation of these pathways culminated in protection of the heart tissue against injury and cell death, thereby explaining the reduction in IFS associated with the administration of 1 μM FTY720 at the onset of reperfusion, as well as 2.5 μM both prior to ischaemia and at the onset of reperfusion. The effect of the pre-ischaemic administration of FTY720 on IFS may be due to the contribution of PP2A activation, which at this dose and during ischaemia and the onset of reperfusion, opposes cardioprotective signalling by de-phosphorylating some of the proteins involved in the mediation of protection.

Theoretically, possible targets for PP2A under these conditions include PKC, PKA, PKB/Akt and ERK p42/p44.^79^ Work done in our laboratory has also implicated PP2A as a negative regulator of PKB/Akt at the onset of reperfusion (unpublished data). These results highlight the importance of the time point of intervention in determining the outcome of I/R. Simultaneously however, FTY720, especially at the higher dose of 2.5 μM, exerted a potent effect on heart rate and contractility of the heart by contributing to an increase in membrane potential and reducing the availability of Ca^2+^ at the myofibrils of the cardiomyocytes. The result of this is a major and profound reduction in cardiac function during reperfusion.

The prescribed dose of FTY720 for recurring MS is 0.5 mg once daily. This translates into a blood concentration of less than 0.5 ng/ml after 96 hours in renal transplant recipients,^80^,^81^ which is less than 1.45 nM. Even though FTY720 in these small concentration ranges exerted a very small and transient effect on heart rate in patients,^80^,^82^ it is still much lower than the concentrations we used. Our study therefore does not address concerns with regard to the current FTY720 treatment regime. Our results are however of potential importance in the context of anticancer therapy, where the administration of relatively high doses of FTY720 becomes relevant, as well as the potential use of FTY720 to limit the development of myocardial I/R injury.^62^

## Conclusion

We have shown that the effects of acute FTY720 treatment are dependent on both the timing of the intervention, as well as the dose at which it is administered. Although FTY720 has the ability to limit IFS, acute pre-ischaemic administration was much less beneficial than reperfusion administration. Increasing the concentration of FTY720, although still reducing IFS development, exerted a profoundly negative effect on postischaemic heart function. More work is needed to describe the mechanism by which acute FTY720 administration at these concentrations exerts its effects on cardiac function, especially in the context of its effects on kinase/phosphatase signalling and Ca^2+^ handling.
